# Synthesis and structure of tris­(2-methyl-1*H*-imidazol-3-ium) 5-carb­oxy­benzene-1,3-di­carboxyl­ate 3,5-di­carb­oxy­benzoate

**DOI:** 10.1107/S2056989025002063

**Published:** 2025-03-11

**Authors:** Lina Maria Asprilla-Herrera, Simone Techert, Jose de Jesus Velazquez-Garcia

**Affiliations:** a Department of Chemistry, Faculty of Natural and Exact Sciences, Universidad del, Valle, Calle 13 No. 100-00, 760042 Cali, Colombia; b Institut für Röntgenphysik, Georg-August-Universität Göttingen, Friedrich-Hund-Platz 1, 37077 Göttingen, Germany; c Deutsches Elektronen-Synchrotron DESY, Notkestr. 85, 22607 Hamburg, Germany; Universidad Nacional Autónoma de México, México

**Keywords:** crystal structure, 2-methyl­imidazole, trimesic acid

## Abstract

The structure of the title salt, 3C_4_H_7_N_2_^+^·C_9_H_5_O_6_^−^·C_9_H_4_O_6_^2−^, **1**, consists of three 2-methyl-imidazolium cations and both a single and a doubly deprotonated form of trimesic acid as anions. A detailed analysis of the bond lengths and angles reveals both differences and similarities between compound **1** and the previously reported 2-methyl-1*H*-imidazol-3-ium 3,5-di­carb­oxy­benzoate structure [Baletska *et a*l. (2023). *Acta Cryst.* E**79**, 1088–109], as well as the neutral counterpart of the ions. Examination of the crystal packing shows the formation of infinite chains by the anions, which, along with the cations, form zigzag planes parallel to the *ab* plane. The packing inter­actions are primarily driven by π–π inter­actions and hydrogen bonding between anions.

## Abstract 2

The structure of a tris­(2-methyl-1*H*-imidazol-3-ium) di­hydrogenetrimesate^−^ mono­hydrogentrimesate^2−^ compound was determined by single-crystal X-ray diffraction. The compound is mixture of protonated and deprotonated mol­ecules.

## 1. Chemical context

Trimesic acid (H_3_btc, or benzene-1,2,3-tri­carb­oxy­lic acid) and 2-meth­ylimidazole (2-mIm) are two well-known organic compounds with a wide range of applications. Trimesic acid, a planar and highly symmetrical trifunctional compound, has been used for self-assembled mol­ecular monolayers and surface functionalization (Ha *et al.*, 2010 ▸; Lin *et al.*, 2023 ▸; Chen *et al.*, 2014 ▸; Korolkov *et al.*, 2012 ▸; MacLeod, 2019 ▸; Iancu *et al.*, 2013 ▸). Additionally, H_3_btc, along with dendrimers based on it, has been employed in biomolecular delivery systems (Salamończyk, 2011 ▸; Mat Yusuf *et al.*, 2017 ▸; Emani *et al.*, 2023 ▸). On the other hand, 2-mIm, a nitro­gen-containing heterocyclic organic compound, is widely used in the preparation of pharmaceuticals, photographic and photothermographic chemicals, dyes and pigments, agricultural chemicals, and in rubber production (Hachuła *et al.*, 2010 ▸; Chan, 2004 ▸). Both H_3_btc and 2-mIm are also well-known ligands in the syntheses of metal–organic frameworks (MOFs), such as HKUST-1 (Chui *et al.*, 1999 ▸), MIL-100 (Férey *et al.*, 2004 ▸), ZIF-8 (Park *et al.*, 2006 ▸), and ZIF-67 (Banerjee *et al.*, 2008 ▸), which have applications in gas adsorption, catalysis, and drug delivery, among others (Zhong *et al.*, 2018*a* ▸,*b* ▸; Zhao *et al.*, 2024 ▸; Huang *et al.*, 2011 ▸; Song *et al.*, 2024 ▸; Abdelhamid, 2021 ▸; Sun *et al.*, 2012 ▸).

In our previous studies, we synthesised hexa­aqua­cobalt bis­(2-methyl-1*H*-imidazol-3-ium) tetra­aqua­bis­(benzene-1,3,5-tri­carboxyl­ato-κ*O*)cobalt (Velazquez-Garcia & Techert, 2022 ▸) and 2-methyl-1*H*-imidazol-3-ium 3,5-di­carb­oxy­benzoate (Baletska *et al.*, 2023 ▸) using 2-mIm and H_3_btc as organic compounds. In this work, we used the same organic compounds to synthesise the title compound, **1**.
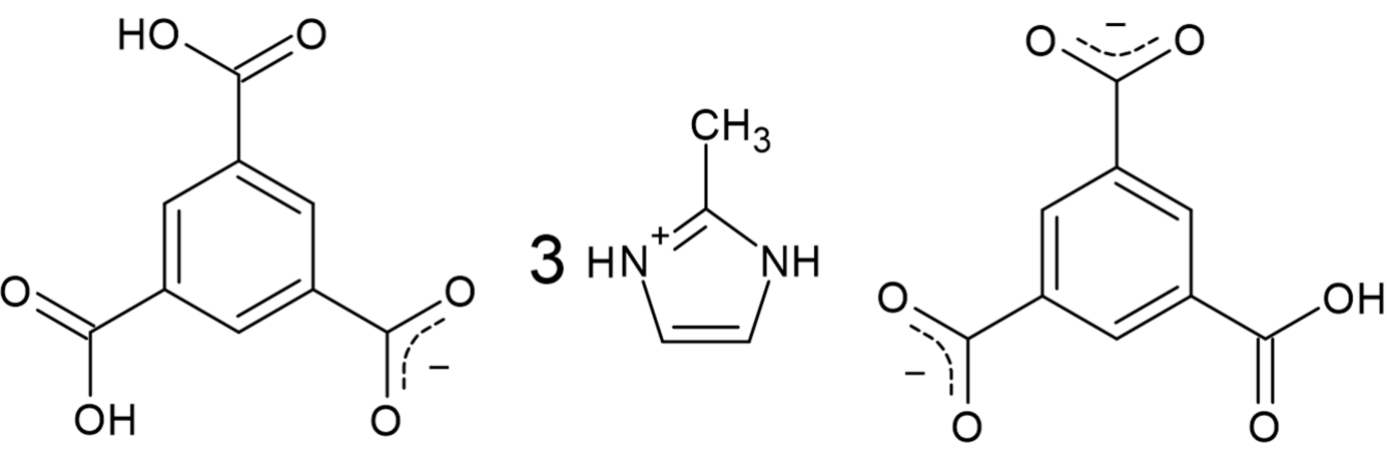


## 2. Structural commentary

**Figure 1:**
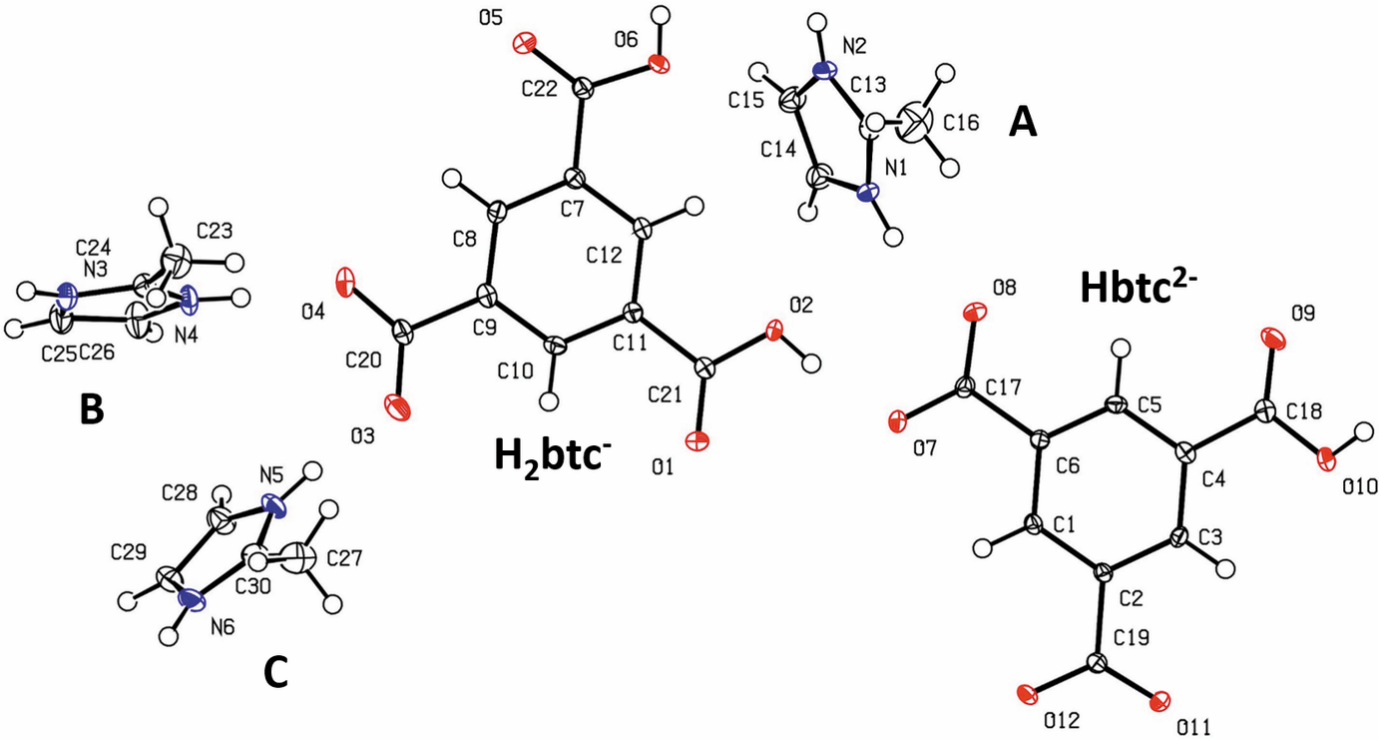
Single-crystal X-ray structure of **1** with displacement ellipsoids drawn at the 50% probability level.

Compound **1** crystallizes with one H_2_btc^−^, one Hbtc^2−^, and three H2-mIm^+^ ions in the asymmetric unit, space group *P*2_1_/*n*. An ellipsoid plot illustrating these ionic species is shown in Fig. 1 ▸. For clarity, the three crystallographically independent cations are labelled as **A**, **B**, and **C** to facilitate their identification.

Table 1 ▸ presents selected bond distances and angles of the H_2_btc^−^ ion, while Table 2 ▸ shows those for the Hbtc^2−^ ion. The shortest bond in the H_2_btc^−^ ion is between C21 and O1 at 1.214 (2) Å, while the longest is between C9 and C20 at 1.519 (2) Å. In the Hbtc^2−^ ion, the shortest bond is C18—O9 at 1.214 (2) Å, and the longest is C6—C17 at 1.510 (2) Å.

The C—C and C—O bond lengths in the H_2_btc^−^ ion range from 1.389 (2) to 1.519 (2) Å and 1.214 (2) to 1.318 (2) Å, respectively. For the Hbtc^2−^ ion, the C—C bond lengths span 1.388 (2) to 1.510 (2) Å, while the C—O bonds range from 1.214 (2) to 1.338 (2) Å. These values are comparable to those in the neutral H_3_btc mol­ecule (Tothadi *et al.*, 2020 ▸), where the C—C bond lengths range from 1.381 (6) to 1.494 (9) Å, and C—O bonds range from 1.229 (5) to 1.303 (5) Å. They are also consistent with the bond lengths observed in the H_2_btc^−^ anion reported in our previous work (Baletska *et al.*, 2023 ▸), and featuring ranges of 1.388 (2)–1.511 (2) Å for C—C bonds and 1.224 (2)–1.320 (2) Å for C—O bonds.

The C—C—C angles in H_2_btc^−^ in **1** range from 118.9 (2) to 120.8 (2)°, while in the Hbtc^2−^ ion, they fall between 119.4 (2) and 120.4 (2)°. These values are comparable to the corresponding angles in H_3_btc [119.0 (4)–121.1 (4)°] and H_2_btc^−^ reported by Baletska *et al.* (2023 ▸) [118.9 (2)–121.4 (4)°]. The O—C—O angles in the H_2_btc^−^ ion in complex **1** span 124.3 (2) to 126.8 (2)°, and in the Hbtc^2−^ ion, they range from 123.2 (2) to 125.4 (2)°. These values are also consistent with those found in neutral H_3_btc [124.4 (4)–125.0 (4)°] and in H_2_btc^−^ from [123.9 (2)–126.1 (2)°; Baletska *et al.*, 2023 ▸].

The main difference between the anions in **1**, the neutral H_3_btc mol­ecule, and the H_2_btc^−^ ion (Baletska *et al.*, 2023 ▸) lies in their torsion angles. In the H_3_btc mol­ecule, the oxygen atoms are nearly coplanar with the aromatic ring, with torsion angles deviating from 0 or 180° by no more than 4.2 (4)°. H_2_btc^−^ (Baletska *et al.*, 2023 ▸) shows a wider deviation range, from 4.2 (2) to 16.6 (2)°. In comparison, the H_2_btc^−^ ion in **1** exhibits inter­mediate values, ranging from 0.6 (2) to 7.0 (2)°, whereas the Hbtc^2−^ ion shows the largest torsion angles, ranging from 12.6 (2) to 17.1 (2)°.

**Figure 2:**
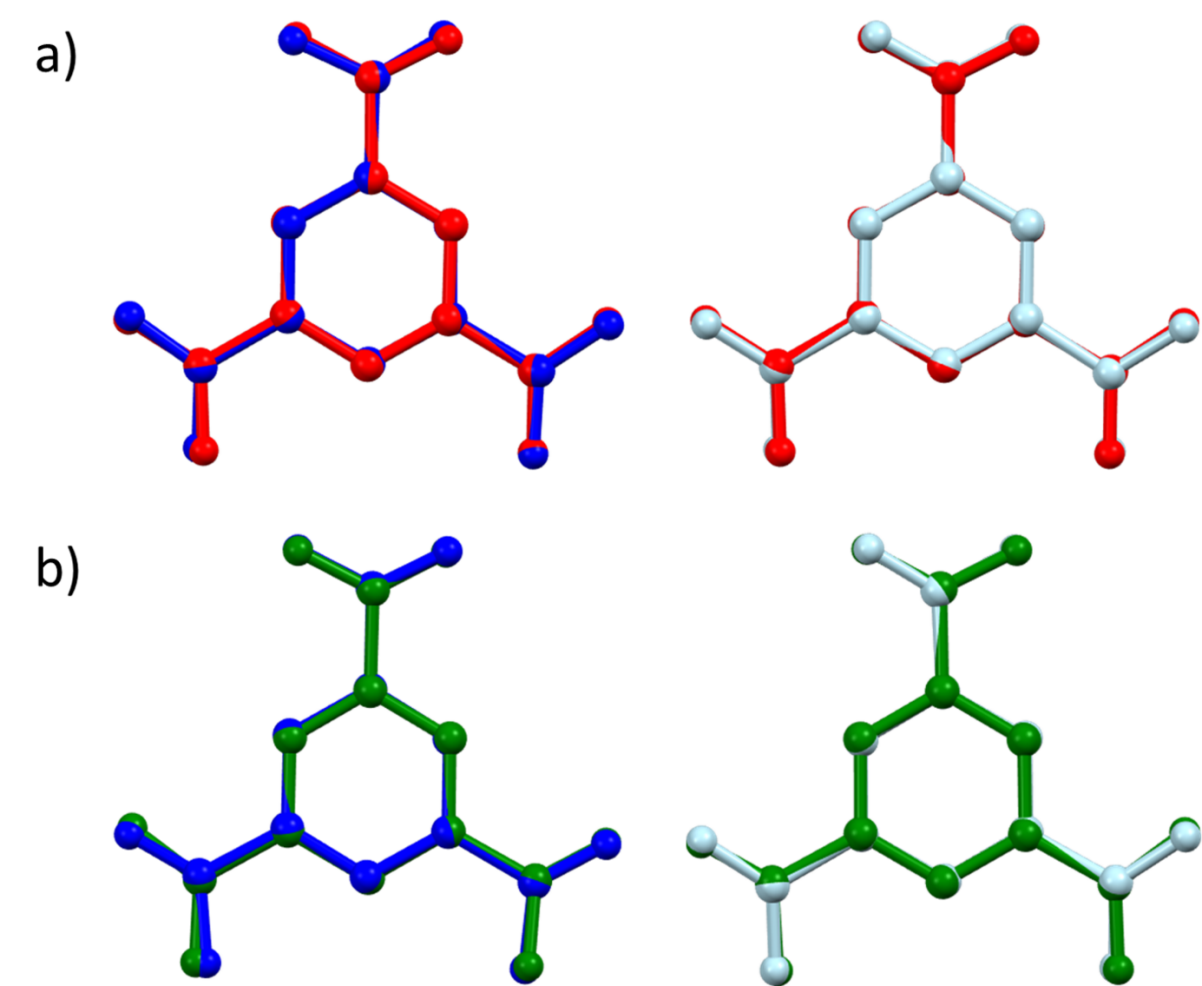
Overlay plot comparing the H_2_btc^−^ (dark blue) and Hbtc^2−^ (light blue) ions in **1** with (*a*) H_3_btc (red; Tothadi *et al.* 2020 ▸) and (*b*) H_2_btc^−^ (green; Baletska *et al.*, 2023 ▸). Hydrogen atoms are omitted for clarity.

These differences are further emphasised through mol­ecular overlays generated using *Mercury* software (Macrae *et al.*, 2020 ▸). The overlays (Fig. 2 ▸) show that the H_2_btc^−^ ion in **1** resembles the neutral H_3_btc more closely (root-mean-square deviation, r.m.s.d. = 0.0683 Å; maximal deviation, max. d. = 0.1257 Å) than the H_2_btc^−^ ion) (r.m.s.d. = 0.1039 Å; max. d. = 0.2189 Å; Baletska *et al.*, 2023 ▸). On the other hand, the Hbtc^2−^ ion in **1** shows a lower resemblance to H_3_btc (r.m.s.d. = 0.1856 Å; max. d. = 0.3985 Å) compared to the H_2_btc^−^ ion (r.m.s.d. = 0.09 Å; max. d. = 0.2344 Å; Baletska *et al.*, 2023 ▸). Note that hydrogen atoms were excluded from the model during the overlay process.

Table 3 ▸ presents selected bond lengths, angles, and torsions for the H2-mIm^+^ cations. The C—C bond distances fall in the range 1.339 (3)–1.483 (3) Å, while the C—N bonds vary from 1.323 (2) to 1.383 (2) Å. These values are comparable to the corresponding distances observed in the neutral 2-mIm^+^ mol­ecule reported by Hachuła *et al.* (2010 ▸) [C—C = 1.367 (1)–1.488 (1) Å, C—N = 1.329 (1)–1.385 (1) Å] and in the H2-mIm^+^ ion reported by Baletska *et al.* (2023 ▸) [C—C = 1.345 (3)–1.481 (3) Å, C—N = 1.327 (2)–1.377 (2) Å].

Imidazole derivatives often exhibit an asymmetry in the two endocyclic N—C bonds (Hachuła *et al.*, 2010 ▸). However, this asymmetry is minimal in all three cations of **1**, with differences between the two N—C bond lengths of 0.001 (3), 0.003 (3), and 0.0 (3) Å for cations **A**, **B**, and **C**, respectively. These values are comparable with the asymmetry found in the H2-mIm^+^ ion [0.008 (3) Å; Baletska *et al.*, 2023 ▸] and are significantly smaller than that reported for the neutral mol­ecule [0.022 (1) Å]. This increased symmetry supports the idea that protonation of the imidazole reduces the disparity between the two endocyclic N—C bonds.

Protonation to an H2-mIm^+^ ion also leads to a more symmetrical heterocyclic ring. In the H2-mIm^+^ ion (Baletska *et al.*, 2023 ▸), this increased symmetry is observed in the C—C—N and N—C—N angles of the heterocyclic ring, which closely approach the ideal penta­gon angle of 108°, with a maximum deviation of 1.6 (2)°. In contrast, the neutral 2-mIm mol­ecule shows a larger deviation of 3.4 (1)°. In compound **1**, the maximum deviations from the ideal angles of a penta­gon are 1.9 (2), 1.9 (2), and 1.7 (2)° for cations **A**, **B**, and **C**, respectively. These values confirm that the protonated imidazole exhibits a more symmetrical ring structure than its neutral counterpart.

**Figure 3:**
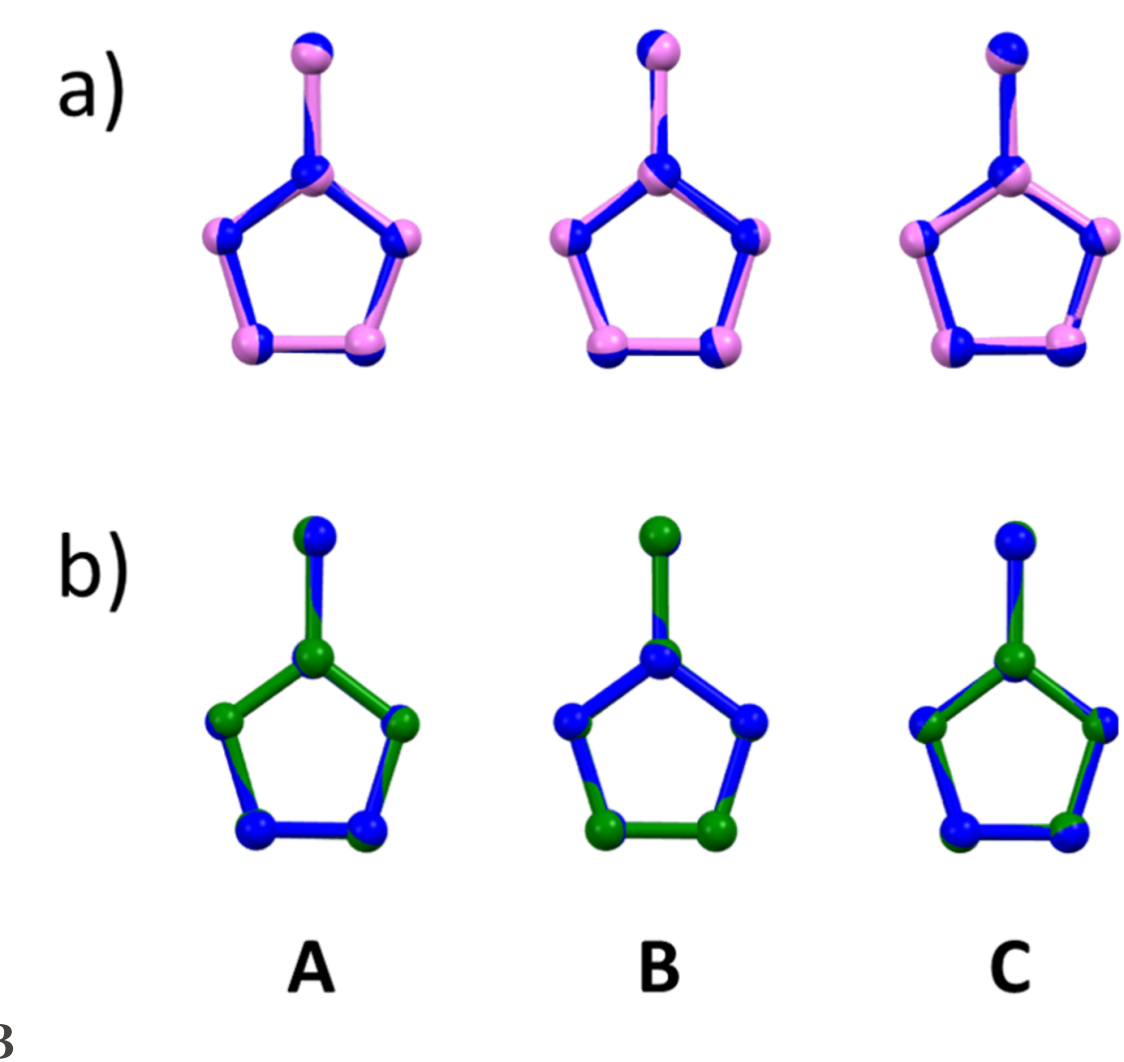
Overlay plot comparing the three H2-mIm^+^ ions (dark blue - **A**, **B** and **C**) in **1** with (*a*) 2-mIm (pink; Hachułaet al., 2010) and (*b*) H2-mIm^+^ ion (green; Baletska *et al.*,, 2023 ▸). Hydrogen atoms are omitted for clarity.

An analysis of the torsion angles in all cations in compound **1** reveals that the methyl group in cation **A** is less coplanar to the ring than in other cations. This is evident from the maximum deviation from 180° of the C—N—C—C^Me^ torsion angles (where C^Me^ represents the carbon from the methyl group). Cation **A** shows a deviation of 2.3 (2)°, while cations **B** and **C** exhibit smaller deviations of 0.9 (2) and 0.3 (2)°, respectively. The deviation in cation **A** is also larger than that observed in the neutral mol­ecule [0.7 (1)°] and the H2-mIm^+^ ion [0.5 (2)°; Baletska *et al.*, 2023 ▸]. The root-mean-squared deviation (r. m. s. d.) and maximal deviation (max. d.) values, calculated by *Mercury* software for the mol­ecular overlays of the three H2-mIm^+^ cations in **1** with the H2-mIm^+^ cation (Baletska *et al.*, 2023 ▸) and the neutral mol­ecule (Fig. 3 ▸), show a greater similarity between the protonated forms compared to the neutral mol­ecule. The r. m. s. d. and max. d. values for the cations of **1** and the protonated H2-mIm^+^ (Baletska *et al.*, 2023 ▸) range from 0.0067 to 0.0140 Å and 0.0092 to 0.0201 Å, respectively, indicating a close resemblance. On the other hand, the values for the neutral mol­ecule are notably higher, ranging from 0.0269 to 0.0297 Å (r.m.s.d.) and 0.0402 to 0.0474 Å (max. d.). In all cases, hydrogen atoms were omitted from the model during the overlay process.

## 3. Supra­molecular features

**Figure 4:**
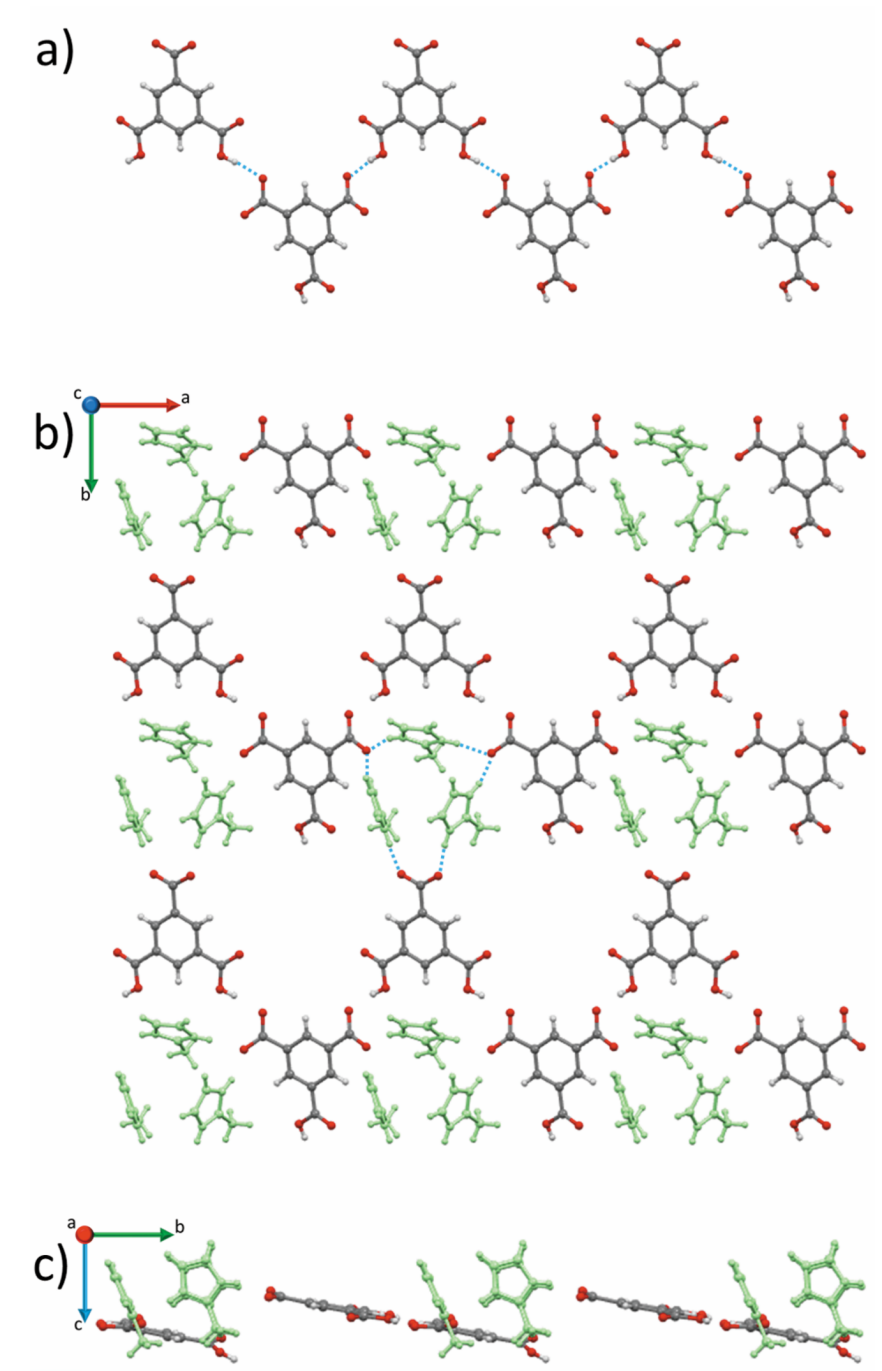
(*a*) View down the *c* axis showing an infinite chain of H_2_btc^−^–Hbtc^2−^ anions running along the *a* axis. A plane formed by the H2-mIm^+^ ions (green) and the H_2_btc^−^-Hbtc^2−^ chains, view down (*b*) the *c* axis and (*c*) the *a* axis.

**Figure 5:**
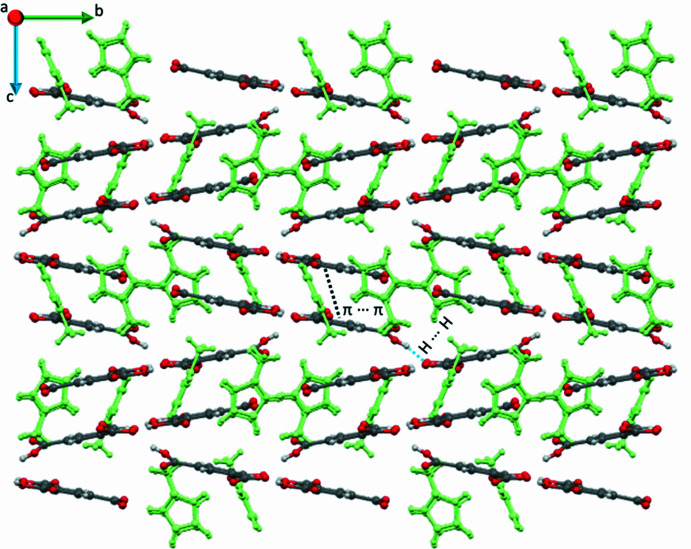
Crystal packing in compound **1** viewed down the *a* axis showing the π–π inter­actions and hydrogen bonding connecting the 2*H*-mim^+^–H_2_btc^−^–Hbtc^2−^ planes that run parallel to the *ab* plane. The H2-mIm^+^ ions are highlighted in green.

The primary inter­molecular inter­action contributing to the crystal packing includes hydrogen bonds between all ions, along with π–π stacking between anions. Table 4 ▸ provides a summary of the hydrogen bonds found within the compound. As shown in Fig. 4 ▸*a*, infinite chains are formed along the *a* axis through hydrogen bonding between H_2_btc^−^ and Hbtc^2−^ anions. These chains are further linked, *via* hydrogen bonding, with all of the cations, forming zigzag planes parallel to the *ab* plane (Fig. 4 ▸*b*,*c*). Each plane inter­acts with two types of neighbouring planes: one with a parallel zigzag pattern, inter­acting *via* π–π stacking between H_2_btc^−^ and Hbtc^2−^ ions [centroid-to-centroid distance of 3.5663 (12) Å, perpendicular distance between planes ∼3.3 Å and offset of 1.249 Å], and another arranged in an anti­parallel configuration, with the zigzag pattern running in the opposite direction. This anti­parallel plane inter­acts *via* hydrogen bonding between Hbtc^2−^ ions (Fig. 5 ▸). Note that the spaces observed in the planes in Fig. 4 ▸*b* are filled by counter-ions from the adjacent planes with a parallel zigzag pattern, ensuring no voids within compound **1**.

**Figure 6:**
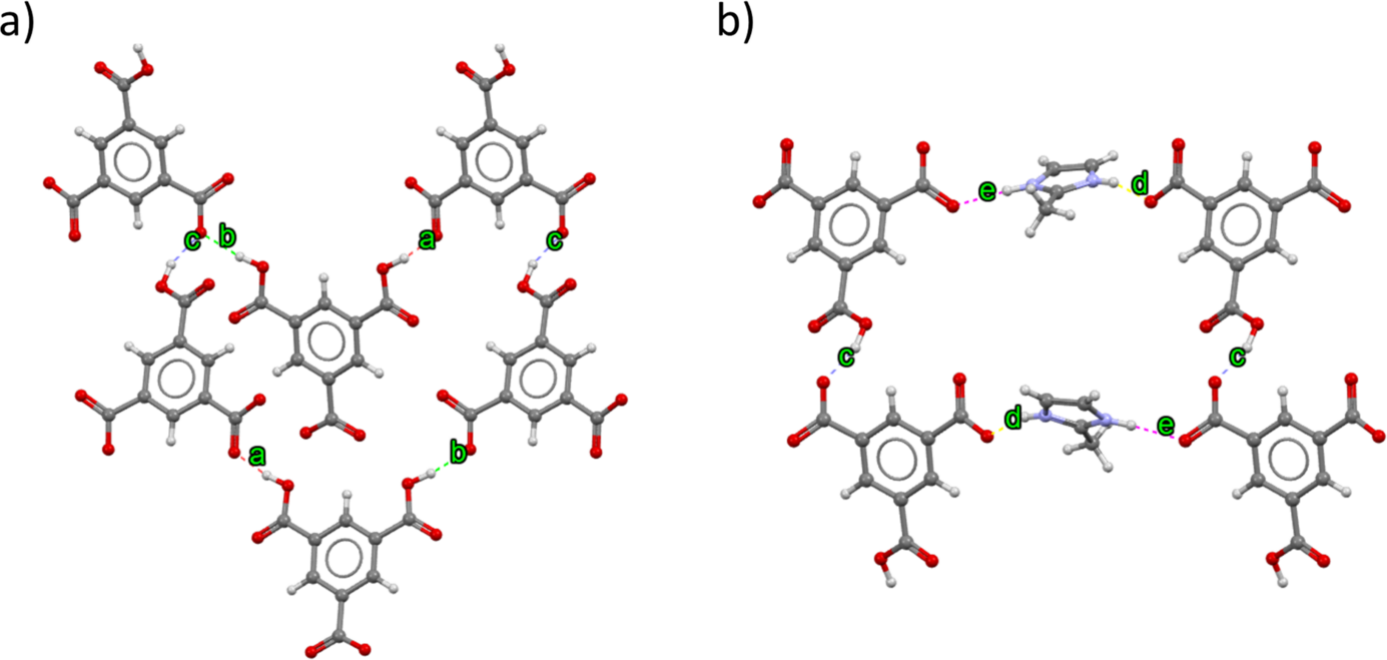
View along the *c* axis showing the formation of hydrogen-bonded ring patterns with the graph-set descriptors: (*a*) 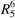
(42) and (*b*) 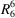
(36).

A graph-set analysis (Etter *et al.*, 1990 ▸; Bernstein *et al.*, 1995 ▸) allows a more detailed examination of the inter­molecular inter­action patterns within **1**. The analysis reveals that **1** contains nine motifs at the first-level graph set, including eight discrete *D*(2) motifs and one chain motif *C*(8), labelled as type *c* in Table 4 ▸. The second-level graph set (Table 5 ▸) reveals a complex network of inter­molecular inter­actions within **1**, featuring various patterns: 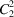
(16) >*a*<*b*, 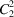
(12) >*d*<*e*, several *D*_3_^3^ such as >*a*>*c*<*a*, >*d*>*c*<*d*, >*e*>*c*<*e*, >*f*>*c*<*f*, >*i*>*c*<I and many *D*_2_^2^, for example >*a*<d, >*a*<*e* and >*a*<*f*. A different pattern, rather than discrete and chain, appears in the third order graph set with formation of the rings 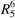
(42) >*a*>*c*〈*b*〉*a*<*c*<*b* (Fig. 6 ▸*a*) and 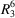
(36) >*c*<*d*>*e*<*c*<*d*>*e* (Fig. 6 ▸*b*).

## 4. Database survey

No reported structures of the title compound were found in the Cambridge Structural Database (CSD version 5.45, update of November 2023; Groom *et al.*, 2016 ▸). The closest to **1** is the previously mentioned structure reported under the refcode LODSUW (Baletska *et al.*, 2023 ▸).

Among the various reported structures containing the H2-mIm^+^ cation, we highlight those with the following refcodes: BEZGEU (Dhanabal *et al.*, 2013 ▸), BOTTEK, BOTTIO, BOTTOU (Meng *et al.*, 2009 ▸), BOTTEK01, BOTTIO01, BOTTOU01, VURBUG, VURCAN, VURFAQ (Callear *et al.*, 2010 ▸), DAMGIL (Hinokimoto *et al.*, 2021 ▸), DOWVUI (Shi *et al.*, 2014 ▸), FAMFIL, FAMFOR, FAMFUX (Zhang & Zhang, 2017 ▸), FETDAK (Aakeröy *et al.*, 2005 ▸), and HILSOL (Qu, 2007 ▸).

Organic compounds containing both H_2_btc^−^ and Hbtc^2^- were found with the refcodes: RAVPOV (Arunachalam *et al.*, 2012 ▸), SADKUE (Fan *et al.*, 2003 ▸), and TUBBAT (Melendez *et al.*, 1996 ▸). Some compounds with low resemblance to the title compound were reported under the refcodes CUMQUX (Basu *et al.*, 2009 ▸), HICSUJ (Lie *et al.*, 2013 ▸), ILELAO (Li & Li, 2016 ▸), JOCBAH (Falek *et al.*, 2019 ▸), LUBGUM, LUBHAT, LUBHEX, LUBHIB, LUBHOH, LUBHUN, LUBJAV (Singh *et al.*, 2015 ▸), SUHRAR (Rajkumar *et al.*, 2020 ▸), YOCSIT (Habib & Janiak, 2008 ▸), and WOGBED (Sosa-Rivadeneyra *et al.*, 2024 ▸).

## 5. Synthesis and crystallization

To obtain the title compound, 800 µl of an ethano­lic solution of 2-mlm (1.57 *M*) was diluted in 20 ml of ethanol, followed by the addition of 1 ml of an ethano­lic solution of H_3_btc (0.12 *M*). The mixture was shaken gently, but no visible changes were observed after 5 min. Crystals of **1** were obtained after 24 h.

## 6. Refinement

Crystal data, data collection, and structure refinement details are summarized in Table 6 ▸. The positions of hydrogen atoms were refined with *U*_iso_(H) = 1.2*U*_eq_(C) for CH. Hydrogen atoms bonded to nitro­gen atoms (N—H) and oxygen atoms (O—H) were treated with free refinement of bond distances and isotropic displacement parameters (*U*_iso_).

## Supplementary Material

Crystal structure: contains datablock(s) I. DOI: 10.1107/S2056989025002063/jq2038sup1.cif

Supporting information file. DOI: 10.1107/S2056989025002063/jq2038Isup2.cml

CCDC reference: 2428811

Additional supporting information:  crystallographic information; 3D view; checkCIF report

## Figures and Tables

**Table 1:** Selected bond lengths (Å), angles (°) and torsion angles (°) of the H_2_btc^−^ anion in **1**

C10—C11	1.392 (2)	C7—C12	1.391 (2)	C8—C9	1.389 (2)
C11—C12	1.393 (2)	C7—C8	1.394 (2)	C9—C10	1.394 (2)
C11—C21	1.499 (2)	C7—C22	1.492 (2)	C9—C20	1.519 (2)
O1—C21	1.214 (2)	O3—C20	1.247 (2)	O5—C22	1.218 (2)
O2—C21	1.303 (2)	O4—C20	1.258 (2)	O6—C22	1.318 (2)
C10—C11—C12	119.68 (15)	C7—C12—C11	119.86 (16)	O1—C21—O2	124.30 (16)
C9—C8—C7	120.68 (15)	C12—C7—C8	119.93 (15)	O3—C20—O4	126.76 (17)
C8—C9—C10	118.98 (16)	C11—C10—C9	120.84 (15)	O5—C22—O6	124.31 (15)
C10—C11—C21—O1	−4.4 (2)	C10—C9—C20—O4	−173.05 (15)	C10—C11—C12—-C7	2.3 (2)
C12—C11—C21—O1	174.23 (16)	C8—C9—C20—O4	5.4 (2)	C12—C7—C8—C9	0.0 (2)
C10—C11—C21—O2	176.85 (15)	C12—C7—C22—O5	−177.56 (16)	C7—C8—C9—C10	1.5 (2)
C12—C11—C21—O2	−4.5 (2)	C8—C7—C22—O5	1.9 (2)	C8—C9—C10—C11	−1.2 (2)
C10—C9—C20—O3	6.0 (2)	C12—C7—C22—O6	1.2 (2)	C8—C7—C12—C11	−1.9 (2)
C8—C9—C20—O3	−175.60 (15)	C8—C7—C22—O6	−179.37 (15)	C9—C10—C11—C12	−0.7 (2)

**Table 2:** Selected bond lengths (Å), angles (°) and torsion angles (°) of the Hbtc^2−^ anion in **1**

C1—C6	1.393 (2)	C2—C3	1.39 (2)	C4—C5	1.392 (2)
C1—C2	1.398 (2)	C3—C4	1.391 (2)	C5—C6	1.388 (2)
C2—C19	1.504 (2)	C4—C18	1.486 (2)	C6—C17	1.510 (2)
O7—C17	1.255 (2)	O9—C18	1.214 (2)	O11—C19	1.2555 (19)
O8—C17	1.2650 (19)	O10—C18	1.338 (2)	O12—C19	1.263 (2)
C2—C3—C4	119.79 (15)	C6—C1—C2	120.38 (16)	O7—C17—O8	125.41 (15)
C6—C5—C4	120.39 (15)	C3—C2—C1	119.79 (14)	O9—C18—O10	123.24 (16)
C3—C4—C5	120.21 (16)	C5—C6—C1	119.38 (15)	O11—C19—O12	124.16 (15)
C1—C6—C17—O7	15.5 (2)	C3—C4—C18—O10	17.1 (2)	C1—C2—C3—C4	2.5 (2)
C5—C6—C17—O7	167.31 (14)	C5—C4—C18—O10	164.63 (14)	C2—C3—C4—C5	−0.5 (2)
C1—C6—C17—O8	163.84 (15)	C1—C2—C19—O11	−163.18 (15)	C2—C1—C6—C5	−0.4 (2)
C5—C6—C17—O8	−13.4 (2)	C3—C2—C19—O11	13.5 (2)	C3—C4—C5—C6	−1.5 (2)
C3—C4—C18—O9	−163.64 (16)	C1—C2—C19—O12	15.8 (2)	C4—C5—C6—C1	1.6 (2)
C5—C4—C18—O9	14.6 (2)	C3—C2—C19—O12	−167.44 (15)	C6—C1—C2—C3	−2.4 (2)

**Table 3:** Selected bond lengths (Å), angles (°) and torsion angles (°) of the H2-mIm^+^ cations in **1**

**A**		**B**		**C**	
C13—C16	1.483 (3)	C23—C24	1.482 (3)	C27—C30	1.482 (3)
C14—C15	1.348 (2)	C25—C26	1.339 (3)	C28—C29	1.346 (3)
N1—C13	1.326 (2)	N3—C24	1.332 (2)	N5—C30	1.330 (2)
N1—C14	1.370 (2)	N3—C25	1.383 (2)	N5—C28	1.380 (2)
N2—C13	1.330 (2)	N4—C24	1.323 (2)	N6—C30	1.335 (2)
N2—C15	1.371 (2)	N4—C26	1.380 (2)	N6—C29	1.380 (2)
C13—N2—C15	109.13 (14)	C28—C29—N6	106.06 (17)	C24—N4—C26	108.48 (16)
C13—N1—C14	109.87 (15)	C29—C28—N5	107.12 (17)	C24—N3—C25	109.08 (15)
C14—C15—N2	107.24 (16)	C30—N5—C28	109.18 (16)	C25—C26—N4	107.85 (16)
C15—C14—N1	106.39 (15)	C30—N6—C29	108.92 (16)	C26—C25—N3	106.35 (17)
N1—C13—N2	107.36 (16)	N4—C24—N3	108.31 (16)	N5—C30—N6	107.91 (16)
N1—C14—C15—N2	0.1 (2)	N3—C25—C26—N4	0.0 (2)	N5—C28—C29—N6	−0.5 (2)
C13—N1—C14—C15	0.4 (2)	C24—N3—C25—C26	0.4 (2)	C30—N5—C28—C29	0.5 (2)
C14—N1—C13—N2	−0.8 (2)	C25—N3—C24—N4	0.7 (2)	C28—N5—C30—N6	−0.2 (2)
C13—N2—C15—C14	−0.5 (2)	C24—N4—C26—C25	−0.5 (2)	C30—N6—C29—C28	0.4 (2)
C15—N2—C13—N1	0.8 (2)	C26—N4—C24—N3	0.7 (2)	C29—N6—C30—N5	0.0 (2)
C14—N1—C13—C16	177.8 (2)	C26—N4—C24—C23	179.13 (18)	C28—N5—C30—C27	−179.94 (17)
C15—N2—C13—C16	−177.7 (2)	C25—N3—C24—C23	179.12 (18)	C29—N6—C30—C27	179.67 (18)

**Table 4:** Hydrogen-bond geometry (Å, °).

	Graph-set descriptor	type	*D*—H	H⋯*A*	*D*⋯*A*	*D*—H⋯*A*
N1—H1*A*⋯O8^V^	*D*(2)	*d*	0.86 (2)	1.911 (18)	2.737 (2)	160.8 (7)
O2—H2⋯O7^i^	*D*(2)	*a*	0.96 (3)	1.57 (2)	2.5222 (19)	170.7 (17)
N2—H2*A*⋯O11^iv^	*D*(2)	*e*	0.88 (3)	1.93 (2)	2.806 (2)	172.5 (13)
N3—H3*A*⋯O11	*D*(2)	*f*	0.935 (19)	1.874 (19)	2.778 (2)	162.1 (18)
N4—H4⋯O4^vi^	*D*(2)	*g*	1.01 (2)	1.59 (2)	2.593 (2)	172.6 (9)
N5—H5*A*⋯O3^vii^	*D*(2)	*h*	1.01 (2)	1.69 (2)	2.655 (2)	159.9 (5)
O6—H6⋯O12^ii^	D(2)	*b*	0.93 (3)	1.69 (2)	2.6189 (19)	171.7 (16)
N6—H6*A*⋯O8	*D*(2)	*i*	0.921 (17)	1.886 (19)	2.800 (2)	170.9 (13)
O10—H10*A*⋯O12^iii^	*C*(8)	*c*	0.93 (3)	1.71 (3)	2.6156 (18)	162 (2)
						
C14—H14⋯O1^v^			0.95	2.52	3.098 (2)	119
C15—H15⋯O10			0.95	2.46	3.280 (2)	144
C15—H15⋯O5^iv^			0.95	2.38	3.038 (2)	126
C25—H25⋯O5			0.95	2.55	3.292 (2)	135
C27—H27*B*⋯O9			0.98	2.41	3.380 (3)	168
C28—H28⋯O9^vii^			0.95	2.39	2.990 (2)	121
C29—H29⋯O1			0.95	2.33	3.108 (2)	138

(i) 1 − *x*, 1 − *y*, 1 − *z*; (ii) 2 − *x*, 1 − *y*, 1 − *z*; (iii) 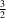 − *x*, 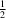 + *y*, 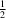 − *z*; (iv) −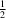 + *x*, 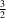 − *y*, −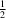 + *z*; (v) 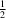 + *x*, 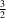 − *y*, −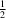 + *z*; (vi) 2 − *x*, 2 − *y*, 1 − *z*; (vii 1 − *x*, 2 − *y*, 1 − *z*.

**Table 5:** Second- and third-level graph sets

	Second-level		Third-level		
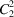 (16)	>*a*<*b*	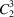 (18)	>*a*>*c*<*b*	*D*_3_^3^(17)	>*d*<*b*<*h*
*D*_3_^3^(17)	>*a*>*c*<*a*	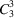 (24)	>*a*<*c*<*b*	*D*_3_^3^(13)	>*e*<*b*<*g*
*D*_2_^2^(5)	>*a*<*d*	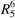 (42)	>*a*>*c*〈*b*〉*a*<*c*<*b*	*D*_3_^3^(13)	>*e*<*b*<*h*
*D*_2_^2^(9)	>*a*<*e*	*D*_3_^3^(17)	>*a*<*c*<*d*	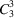 (16)	>*b*<*f*>*g*
*D*_2_^2^(9)	>*a*<*f*	*D*_3_^3^(17)	>*a*>*c*<*d*	*D*_3_^3^(13)	>*f*<*b*<*h*
*D*_2_^2^(10)	>*g*>*a*	*D*_3_^3^(13)	>*a*>*c*<*e*	*D*_3_^3^(17)	>*g*>*b*<*i*
*D*_2_^2^(10)	>*h*>*a*	*D*_3_^3^(17)	>*a*<*c*<*e*	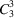 (20)	>*b*〈*i*〉*h*
*D*_2_^2^(5)	>*a*<*i*	*D*_3_^3^(13)	>*a*>*c*<*f*	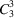 (16)	>*c*<*e*>*d*
*D*_2_^3^ (11)	>*b*>*c*<*b*	*D*_3_^3^(17)	>*a*<*c*<*f*	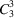 (20)	>*c*<*d*>*e*
*D*_2_^2^(9)	>*b*<*d*	*D*_3_^3^(17)	>*a*<*c*<*i*	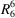 (36)	>*c*<*d*>*e*<*c*<*d*>*e*
*D*_2_^2^(5)	>*b*<*e*	*D*_3_^3^(17)	>*a*>*c*<*i*	*D*_3_^3^(13)	>*d*>*c*<*f*
*D*_2_^2^(5)	>*b*<*f*	*D*_3_^3^(13)	>*d*<*a*<*g*	*D*_3_^3^(17)	>*d*<*c*<*f*
*D*_2_^2^(10)	>*g*>*b*	*D*_3_^3^(13)	>*d*<*a*<*h*	*D*_3_^3^(17)	>*d*<*c*<i
*D*_2_^2^(10)	>*h*>*b*	*D*_3_^3^(17)	>*e*<*a*<*g*	*D*_3_^3^(17)	>*d*>*c*<i
*D*_2_^2^(9)	>*b*<*i*	*D*_3_^3^(17)	>*e*<*a*<*h*	*D*_3_^3^(13)	>*e*<*c*<*f*
*D*_3_^3^(17)	>*d*>*c*<*d*	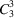 (20)	>*a*<*f*>*g*	*D*_3_^3^(13)	>*e*>*c*<*f*
*D*_3_^3^(13)	>*e*>*c*<*e*	*D*_3_^3^(17)	>*f*<*a*<*h*	*D*_3_^3^(13)	>*e*<*c*<*i*
*D*_3_^3^(13)	>*f*>*c*<*f*	*D*_3_^3^(13)	>*g*>*a*<*i*	*D*_3_^3^(17)	>*e*>*c*<*i*
*D*_3_^3^(17)	>*i*>*c*<*i*	C_3_^3^(16)	>*a*〈*i*〉*h*	*D*_3_^3^(13)	>*f*<*c*<*i*
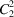 (12)	>*d*<*e*	*D*_2_^3^(11)	>*b*<*c*<*d*	*D*_3_^3^(17)	>*f*>*c*<*i*
*D*_2_^2^(9)	>*d*<*f*	*D*_3_^3^(17)	>*b*>*c*<*d*	*D*_3_^3^(14)	>*d*<*f*>*g*
*D*_1_^2^(3)	>*d*<*i*	*D*_2_^3^(11)	>*b*<*c*<*e*	*D*_2_^3^(8)	>*d*〈*i*〉*h*
*D*_1_^2^(3)	>*e*<*f*	*D*_3_^3^(13)	>*b*>*c*<*e*	*D*_2_^3^(8)	>*e*<*f*>*g*
*D*_2_^2^(9)	>*e*<*i*	*D*_2_^3^(11)	>*b*<*c*<*f*	*D*_3_^3^(14)	>*e*〈*i*〉*h*
*D*_2_^2^(7)	<*f*>*g*	*D*_3_^3^(13)	>*b*>*c*<*f*	*D*_3_^3^(10)	>*h*<g>*f*
*D*_2_^2^(9)	>*f*<*i*	*D*_2_^3^(11)	>*b*<*c*<*i*	*D*_3_^3^(14)	>i<*f*>g
*D*_2_^2^(5)	>*g*<*h*	*D*_3_^3^(17)	>*b*>*c*<*i*	*D*_3_^3^(14)	>*f*〈*i*〉*h*
*D*_2_^2^(7)	<*h*>*i*	*D*_3_^3^ (17)	>*d*<*b*<*g*	*D*_3_^3^(10)	>*g*<*h*>*i*

**Table 6:** Experimental details

Crystal data	
Chemical formula	3C_4_H_7_N_2_^+^·C_9_H_4_O_6_^2−^·C_9_H_5_O_6_^−^
*M*_r_	666.60
Crystal system, space group	Monoclinic, *P*2_1_/*n*
Temperature (K)	100
*a*, *b*, *c* (Å)	14.172 (3), 15.902 (3), 14.644 (3)
β (°)	110.46 (3)
*V* (Å^3^)	3092.0 (12)
*Z*	4
Radiation type	Mo *K*α
μ (mm^−1^)	0.11
Crystal size (mm)	0.08 × 0.07 × 0.05

Data collection	
Diffractometer	Bruker P4
Absorption correction	Multi-scan (*SADABS*; Krause *et al.*, 2015 ▸)
*T*_min_, *T*_max_	0.695, 0.746
No. of measured, independent and observed [*I* > 2σ(*I*)] reflections	36740, 7127, 5287
*R*_int_	0.052
(sin θ/λ)_max_ (Å^−1^)	0.651

Refinement	
*R*[*F*^2^ > 2σ(*F*^2^)], *wR*(*F*^2^), *S*	0.045, 0.119, 1.05
No. of reflections	7127
No. of parameters	457
H-atom treatment	H atoms treated by a mixture of independent and constrained refinement
Δρ_max_, Δρ_min_ (e Å^−3^)	0.35, −0.27

Computer programs: *APEX2* and *SAINT* (Bruker, 2016 ▸), *SHELXT2018/2* (Sheldrick, 2015*a* ▸), *SHELXL2018/3* (Sheldrick, 2015*b* ▸) and *OLEX2* (Dolomanov *et al.*, 2009 ▸).

## supplementary crystallographic information

### Tris(2-methyl-1H-imidazol-3-ium) 5-carboxybenzene-1,3-dicarboxylate 3,5-dicarboxybenzoate Crystal data

**Table:**

3C_4_H_7_N_2_^+^·C_9_H_4_O_6_^2^^−^·C_9_H_5_O_6_^−^	*F*(000) = 1392
*M**_r_* = 666.60	*D*_x_ = 1.432 Mg m^−^^3^
Monoclinic, *P*2_1_/*n*	Mo *K*α radiation, λ = 0.71073 Å
*a* = 14.172 (3) Å	Cell parameters from 5589 reflections
*b* = 15.902 (3) Å	θ = 2.5–26.9°
*c* = 14.644 (3) Å	µ = 0.11 mm^−^^1^
β = 110.46 (3)°	*T* = 100 K
*V* = 3092.0 (12) Å^3^	Irregular, clear light colourless
*Z* = 4	0.08 × 0.07 × 0.05 mm

### Tris(2-methyl-1H-imidazol-3-ium) 5-carboxybenzene-1,3-dicarboxylate 3,5-dicarboxybenzoate Data collection

**Table:**

Bruker P4 diffractometer	*R*_int_ = 0.052
ω scans	θ_max_ = 27.6°, θ_min_ = 2.0°
Absorption correction: multi-scan (SADABS; Krause *et al.*, 2015)	*h* = −18→18
*T*_min_ = 0.695, *T*_max_ = 0.746	*k* = −17→20
36740 measured reflections	*l* = −19→19
7127 independent reflections	Standard reflections: not measured; every not measured reflections
5287 reflections with *I* > 2σ(*I*)	intensity decay: not measured

### Tris(2-methyl-1H-imidazol-3-ium) 5-carboxybenzene-1,3-dicarboxylate 3,5-dicarboxybenzoate Refinement

**Table:**

Refinement on *F*^2^	Primary atom site location: dual
Least-squares matrix: full	Hydrogen site location: inferred from neighbouring sites
*R*[*F*^2^ > 2σ(*F*^2^)] = 0.045	H atoms treated by a mixture of independent and constrained refinement
*wR*(*F*^2^) = 0.119	*w* = 1/[σ^2^(*F*_o_^2^) + (0.0496*P*)^2^ + 1.5554*P*] where *P* = (*F*_o_^2^ + 2*F*_c_^2^)/3
*S* = 1.05	(Δ/σ)_max_ < 0.001
7127 reflections	Δρ_max_ = 0.35 e Å^−^^3^
457 parameters	Δρ_min_ = −0.27 e Å^−^^3^
0 restraints	

### Tris(2-methyl-1H-imidazol-3-ium) 5-carboxybenzene-1,3-dicarboxylate 3,5-dicarboxybenzoate Special details

**Table:**

Geometry. All esds (except the esd in the dihedral angle between two l.s. planes) are estimated using the full covariance matrix. The cell esds are taken into account individually in the estimation of esds in distances, angles and torsion angles; correlations between esds in cell parameters are only used when they are defined by crystal symmetry. An approximate (isotropic) treatment of cell esds is used for estimating esds involving l.s. planes.
Refinement. Hydrogen atoms bonded to nitrogen and oxygen were refined with free isotropic displacement parameters and bond lengths (AFIX 44/148)

### Tris(2-methyl-1H-imidazol-3-ium) 5-carboxybenzene-1,3-dicarboxylate 3,5-dicarboxybenzoate Fractional atomic coordinates and isotropic or equivalent isotropic displacement parameters (Å^2^)

**Table:**

	*x*	*y*	*z*	*U*_iso_*/*U*_eq_	
O1	0.53578 (9)	0.64000 (8)	0.61849 (11)	0.0259 (3)	
O2	0.61872 (9)	0.52011 (8)	0.62429 (10)	0.0220 (3)	
H2	0.5602 (19)	0.4963 (7)	0.6328 (19)	0.053 (8)*	
O3	0.68318 (10)	0.90396 (8)	0.54264 (10)	0.0241 (3)	
O4	0.83140 (9)	0.89827 (8)	0.52006 (10)	0.0234 (3)	
O5	1.02817 (8)	0.63301 (8)	0.58809 (9)	0.0180 (3)	
O6	0.95050 (9)	0.51574 (8)	0.60965 (10)	0.0190 (3)	
H6	1.0140 (19)	0.4921 (7)	0.6229 (19)	0.051 (7)*	
C7	0.86039 (11)	0.64249 (11)	0.58751 (11)	0.0121 (3)	
C8	0.85460 (12)	0.72890 (11)	0.57047 (11)	0.0129 (3)	
H8	0.910200	0.757596	0.562900	0.015*	
C9	0.76831 (12)	0.77347 (11)	0.56445 (11)	0.0128 (3)	
C10	0.68832 (11)	0.73080 (11)	0.57804 (11)	0.0124 (3)	
H10	0.629504	0.760943	0.575260	0.015*	
C11	0.69353 (11)	0.64458 (11)	0.59565 (11)	0.0119 (3)	
C12	0.77922 (11)	0.60010 (11)	0.59865 (11)	0.0121 (3)	
H12	0.782241	0.540944	0.608288	0.015*	
C20	0.76001 (12)	0.86677 (11)	0.54084 (12)	0.0152 (4)	
C21	0.60758 (12)	0.60123 (11)	0.61338 (12)	0.0140 (3)	
C22	0.95475 (12)	0.59710 (11)	0.59439 (12)	0.0137 (3)	
O7	0.52944 (8)	0.55672 (8)	0.35759 (9)	0.0170 (3)	
O8	0.45915 (8)	0.67838 (8)	0.37705 (9)	0.0168 (3)	
O9	0.60022 (9)	0.94414 (8)	0.30521 (9)	0.0191 (3)	
O10	0.73044 (8)	0.93889 (8)	0.25158 (9)	0.0168 (3)	
H10A	0.7044 (12)	0.9897 (17)	0.2214 (19)	0.054 (8)*	
O11	0.95291 (8)	0.68773 (7)	0.35827 (8)	0.0146 (3)	
O12	0.87752 (8)	0.56353 (7)	0.34820 (9)	0.0168 (3)	
C1	0.69841 (11)	0.64122 (11)	0.34545 (11)	0.0110 (3)	
H1	0.704556	0.582377	0.357238	0.013*	
C2	0.77796 (11)	0.68585 (10)	0.33282 (11)	0.0105 (3)	
C3	0.76750 (11)	0.77131 (10)	0.31188 (11)	0.0114 (3)	
H3	0.820330	0.801448	0.300719	0.014*	
C4	0.67944 (12)	0.81258 (11)	0.30732 (11)	0.0121 (3)	
C5	0.60164 (11)	0.76843 (11)	0.32292 (11)	0.0120 (3)	
H5	0.542357	0.797249	0.321299	0.014*	
C6	0.61016 (11)	0.68255 (10)	0.34084 (11)	0.0108 (3)	
C17	0.52617 (11)	0.63554 (11)	0.35966 (11)	0.0120 (3)	
C18	0.66564 (12)	0.90441 (11)	0.28876 (12)	0.0140 (3)	
C19	0.87669 (11)	0.64296 (11)	0.34702 (11)	0.0116 (3)	
N1	0.81021 (10)	0.86622 (9)	−0.05350 (11)	0.0158 (3)	
H1A	0.8634 (15)	0.86270 (13)	−0.0687 (4)	0.036 (6)*	
N2	0.65710 (10)	0.85634 (9)	−0.06081 (11)	0.0169 (3)	
H2A	0.5919 (19)	0.8450 (3)	−0.0820 (6)	0.047 (7)*	
C13	0.71886 (12)	0.84187 (12)	−0.10972 (13)	0.0180 (4)	
C14	0.80713 (13)	0.89782 (12)	0.03242 (13)	0.0191 (4)	
H14	0.862098	0.919778	0.085000	0.023*	
C15	0.71066 (13)	0.89158 (12)	0.02759 (13)	0.0202 (4)	
H15	0.684631	0.908466	0.076363	0.024*	
C16	0.69127 (17)	0.80710 (16)	−0.20962 (16)	0.0405 (6)	
H16A	0.652114	0.848892	−0.256907	0.061*	
H16B	0.650842	0.756108	−0.215027	0.061*	
H16C	0.752612	0.793341	−0.222948	0.061*	
N3	1.04278 (11)	0.82926 (10)	0.46433 (11)	0.0190 (3)	
H3A	1.0001 (13)	0.7868 (13)	0.4287 (11)	0.054 (8)*	
N4	1.12129 (11)	0.94728 (10)	0.50099 (11)	0.0194 (3)	
H4	1.1453 (6)	1.0059 (16)	0.49572 (18)	0.051 (7)*	
C23	1.00047 (15)	0.93620 (14)	0.33006 (14)	0.0280 (5)	
H23A	0.928518	0.941715	0.319353	0.042*	
H23B	1.009719	0.896478	0.282717	0.042*	
H23C	1.027755	0.991192	0.321944	0.042*	
C24	1.05406 (13)	0.90477 (11)	0.43016 (13)	0.0177 (4)	
C25	1.10444 (14)	0.82389 (12)	0.56105 (14)	0.0231 (4)	
H25	1.111071	0.777215	0.603334	0.028*	
C26	1.15295 (15)	0.89749 (12)	0.58330 (14)	0.0253 (4)	
H26	1.200691	0.912727	0.644847	0.030*	
N5	0.38559 (11)	0.94219 (10)	0.51325 (11)	0.0194 (3)	
H5A	0.3614 (7)	1.0020 (16)	0.50764 (19)	0.054 (8)*	
N6	0.43032 (11)	0.82074 (10)	0.47674 (12)	0.0203 (3)	
H6A	0.4446 (4)	0.7773 (13)	0.4420 (10)	0.049 (7)*	
C27	0.37791 (15)	0.92675 (15)	0.34081 (14)	0.0313 (5)	
H27A	0.343417	0.882455	0.294699	0.047*	
H27B	0.441827	0.940409	0.332440	0.047*	
H27C	0.335313	0.977045	0.328545	0.047*	
C28	0.41150 (14)	0.89349 (12)	0.59641 (14)	0.0223 (4)	
H28	0.410189	0.910375	0.658189	0.027*	
C29	0.43899 (13)	0.81759 (12)	0.57354 (13)	0.0213 (4)	
H29	0.460320	0.770711	0.616009	0.026*	
C30	0.39781 (13)	0.89704 (12)	0.44175 (13)	0.0195 (4)	

### Tris(2-methyl-1H-imidazol-3-ium) 5-carboxybenzene-1,3-dicarboxylate 3,5-dicarboxybenzoate Atomic displacement parameters (Å^2^)

**Table:**

	*U*^11^	*U*^22^	*U*^33^	*U*^12^	*U*^13^	*U*^23^
O1	0.0182 (6)	0.0143 (7)	0.0524 (9)	0.0038 (5)	0.0212 (6)	0.0053 (6)
O2	0.0198 (6)	0.0087 (7)	0.0457 (8)	−0.0005 (5)	0.0219 (6)	0.0028 (6)
O3	0.0297 (7)	0.0136 (7)	0.0334 (7)	0.0068 (5)	0.0167 (6)	0.0054 (6)
O4	0.0255 (6)	0.0112 (7)	0.0367 (8)	−0.0038 (5)	0.0149 (6)	0.0028 (6)
O5	0.0141 (5)	0.0155 (7)	0.0271 (7)	0.0002 (5)	0.0105 (5)	0.0017 (5)
O6	0.0120 (5)	0.0089 (7)	0.0361 (7)	0.0021 (5)	0.0083 (5)	0.0016 (5)
C7	0.0131 (7)	0.0112 (9)	0.0118 (7)	0.0003 (6)	0.0039 (6)	−0.0007 (6)
C8	0.0134 (7)	0.0120 (9)	0.0137 (8)	−0.0024 (6)	0.0054 (6)	−0.0006 (7)
C9	0.0156 (7)	0.0103 (9)	0.0117 (7)	−0.0005 (6)	0.0037 (6)	−0.0003 (6)
C10	0.0117 (7)	0.0121 (9)	0.0132 (7)	0.0019 (6)	0.0042 (6)	−0.0017 (6)
C11	0.0124 (7)	0.0110 (9)	0.0127 (7)	0.0003 (6)	0.0048 (6)	0.0008 (6)
C12	0.0141 (7)	0.0088 (9)	0.0133 (7)	0.0001 (6)	0.0046 (6)	0.0001 (6)
C20	0.0198 (8)	0.0107 (9)	0.0156 (8)	−0.0004 (7)	0.0066 (6)	−0.0006 (7)
C21	0.0144 (7)	0.0108 (9)	0.0176 (8)	0.0004 (6)	0.0066 (6)	−0.0004 (7)
C22	0.0145 (7)	0.0114 (9)	0.0153 (8)	0.0001 (6)	0.0051 (6)	−0.0008 (7)
O7	0.0164 (6)	0.0090 (7)	0.0297 (7)	−0.0022 (5)	0.0131 (5)	−0.0004 (5)
O8	0.0149 (5)	0.0150 (7)	0.0255 (6)	0.0011 (5)	0.0134 (5)	0.0003 (5)
O9	0.0242 (6)	0.0121 (7)	0.0263 (7)	0.0059 (5)	0.0155 (5)	0.0020 (5)
O10	0.0162 (6)	0.0102 (7)	0.0252 (7)	0.0003 (5)	0.0086 (5)	0.0046 (5)
O11	0.0092 (5)	0.0118 (6)	0.0223 (6)	−0.0014 (4)	0.0050 (5)	−0.0006 (5)
O12	0.0129 (5)	0.0075 (6)	0.0304 (7)	0.0010 (5)	0.0080 (5)	−0.0024 (5)
C1	0.0130 (7)	0.0089 (9)	0.0112 (7)	−0.0005 (6)	0.0045 (6)	−0.0009 (6)
C2	0.0112 (7)	0.0086 (9)	0.0118 (7)	−0.0002 (6)	0.0041 (6)	−0.0020 (6)
C3	0.0105 (7)	0.0111 (9)	0.0131 (7)	−0.0027 (6)	0.0047 (6)	−0.0006 (6)
C4	0.0143 (7)	0.0101 (9)	0.0119 (7)	0.0012 (6)	0.0044 (6)	0.0004 (6)
C5	0.0114 (7)	0.0127 (9)	0.0130 (7)	0.0021 (6)	0.0058 (6)	−0.0001 (6)
C6	0.0117 (7)	0.0101 (9)	0.0118 (7)	−0.0004 (6)	0.0055 (6)	−0.0008 (6)
C17	0.0132 (7)	0.0126 (9)	0.0115 (7)	−0.0011 (6)	0.0060 (6)	0.0010 (6)
C18	0.0151 (7)	0.0120 (9)	0.0145 (8)	−0.0007 (6)	0.0046 (6)	0.0000 (7)
C19	0.0121 (7)	0.0108 (9)	0.0122 (7)	0.0000 (6)	0.0046 (6)	−0.0010 (6)
N1	0.0130 (6)	0.0137 (8)	0.0243 (8)	0.0002 (6)	0.0111 (6)	0.0004 (6)
N2	0.0105 (6)	0.0143 (8)	0.0263 (8)	−0.0011 (6)	0.0071 (6)	0.0015 (6)
C13	0.0175 (8)	0.0144 (10)	0.0233 (9)	0.0017 (7)	0.0088 (7)	−0.0002 (7)
C14	0.0162 (8)	0.0208 (10)	0.0194 (9)	0.0005 (7)	0.0051 (7)	−0.0016 (7)
C15	0.0200 (8)	0.0229 (11)	0.0210 (9)	0.0012 (7)	0.0114 (7)	−0.0002 (8)
C16	0.0375 (12)	0.0498 (16)	0.0311 (12)	0.0013 (11)	0.0079 (9)	−0.0155 (11)
N3	0.0185 (7)	0.0134 (8)	0.0254 (8)	−0.0034 (6)	0.0081 (6)	−0.0021 (6)
N4	0.0237 (7)	0.0116 (8)	0.0232 (8)	−0.0034 (6)	0.0087 (6)	−0.0001 (6)
C23	0.0291 (10)	0.0294 (12)	0.0227 (10)	−0.0011 (9)	0.0055 (8)	0.0049 (8)
C24	0.0187 (8)	0.0143 (10)	0.0225 (9)	−0.0010 (7)	0.0102 (7)	−0.0013 (7)
C25	0.0250 (9)	0.0177 (10)	0.0246 (9)	−0.0021 (8)	0.0061 (7)	0.0048 (8)
C26	0.0306 (10)	0.0203 (11)	0.0203 (9)	−0.0054 (8)	0.0030 (8)	0.0027 (8)
N5	0.0235 (7)	0.0125 (8)	0.0245 (8)	0.0036 (6)	0.0113 (6)	−0.0008 (6)
N6	0.0194 (7)	0.0152 (9)	0.0276 (8)	0.0025 (6)	0.0096 (6)	−0.0050 (7)
C27	0.0305 (10)	0.0392 (14)	0.0256 (10)	0.0070 (9)	0.0115 (8)	0.0062 (9)
C28	0.0266 (9)	0.0191 (11)	0.0226 (9)	0.0031 (8)	0.0105 (7)	−0.0009 (8)
C29	0.0222 (8)	0.0171 (10)	0.0247 (9)	0.0045 (7)	0.0083 (7)	0.0019 (8)
C30	0.0165 (8)	0.0196 (10)	0.0234 (9)	0.0021 (7)	0.0082 (7)	−0.0009 (8)

### Tris(2-methyl-1H-imidazol-3-ium) 5-carboxybenzene-1,3-dicarboxylate 3,5-dicarboxybenzoate Geometric parameters (Å, º)

**Table:**

O1—C21	1.214 (2)	N1—H1A	0.86 (2)
O2—H2	0.96 (3)	N1—C13	1.326 (2)
O2—C21	1.303 (2)	N1—C14	1.370 (2)
O3—C20	1.247 (2)	N2—H2A	0.89 (3)
O4—C20	1.258 (2)	N2—C13	1.330 (2)
O5—C22	1.218 (2)	N2—C15	1.371 (2)
O6—H6	0.93 (3)	C13—C16	1.483 (3)
O6—C22	1.318 (2)	C14—H14	0.9500
C7—C8	1.394 (2)	C14—C15	1.348 (2)
C7—C12	1.391 (2)	C15—H15	0.9500
C7—C22	1.492 (2)	C16—H16A	0.9800
C8—H8	0.9500	C16—H16B	0.9800
C8—C9	1.389 (2)	C16—H16C	0.9800
C9—C10	1.394 (2)	N3—H3A	0.93 (3)
C9—C20	1.519 (2)	N3—C24	1.332 (2)
C10—H10	0.9500	N3—C25	1.383 (2)
C10—C11	1.392 (2)	N4—H4	1.00 (3)
C11—C12	1.393 (2)	N4—C24	1.323 (2)
C11—C21	1.499 (2)	N4—C26	1.380 (2)
C12—H12	0.9500	C23—H23A	0.9800
O7—C17	1.255 (2)	C23—H23B	0.9800
O8—C17	1.2650 (19)	C23—H23C	0.9800
O9—C18	1.214 (2)	C23—C24	1.482 (3)
O10—H10A	0.93 (3)	C25—H25	0.9500
O10—C18	1.338 (2)	C25—C26	1.339 (3)
O11—C19	1.2555 (19)	C26—H26	0.9500
O12—C19	1.263 (2)	N5—H5A	1.01 (3)
C1—H1	0.9500	N5—C28	1.380 (2)
C1—C2	1.398 (2)	N5—C30	1.330 (2)
C1—C6	1.393 (2)	N6—H6A	0.92 (3)
C2—C3	1.390 (2)	N6—C29	1.380 (2)
C2—C19	1.504 (2)	N6—C30	1.335 (2)
C3—H3	0.9500	C27—H27A	0.9800
C3—C4	1.391 (2)	C27—H27B	0.9800
C4—C5	1.392 (2)	C27—H27C	0.9800
C4—C18	1.486 (2)	C27—C30	1.482 (3)
C5—H5	0.9500	C28—H28	0.9500
C5—C6	1.388 (2)	C28—C29	1.346 (3)
C6—C17	1.510 (2)	C29—H29	0.9500
			
C21—O2—H2	109.5	C13—N2—H2A	125.4
C22—O6—H6	109.5	C13—N2—C15	109.13 (14)
C8—C7—C22	118.97 (14)	C15—N2—H2A	125.4
C12—C7—C8	119.95 (15)	N1—C13—N2	107.36 (16)
C12—C7—C22	121.07 (15)	N1—C13—C16	125.80 (17)
C7—C8—H8	119.7	N2—C13—C16	126.82 (17)
C9—C8—C7	120.64 (15)	N1—C14—H14	126.8
C9—C8—H8	119.7	C15—C14—N1	106.39 (15)
C8—C9—C10	118.98 (16)	C15—C14—H14	126.8
C8—C9—C20	120.62 (15)	N2—C15—H15	126.4
C10—C9—C20	120.39 (15)	C14—C15—N2	107.24 (16)
C9—C10—H10	119.6	C14—C15—H15	126.4
C11—C10—C9	120.84 (15)	C13—C16—H16A	109.5
C11—C10—H10	119.6	C13—C16—H16B	109.5
C10—C11—C12	119.68 (15)	C13—C16—H16C	109.5
C10—C11—C21	119.43 (14)	H16A—C16—H16B	109.5
C12—C11—C21	120.88 (15)	H16A—C16—H16C	109.5
C7—C12—C11	119.86 (16)	H16B—C16—H16C	109.5
C7—C12—H12	120.1	C24—N3—H3A	125.5
C11—C12—H12	120.1	C24—N3—C25	108.95 (15)
O3—C20—O4	126.72 (17)	C25—N3—H3A	125.5
O3—C20—C9	117.21 (15)	C24—N4—H4	125.8
O4—C20—C9	116.06 (15)	C24—N4—C26	108.48 (16)
O1—C21—O2	124.30 (16)	C26—N4—H4	125.8
O1—C21—C11	121.75 (16)	H23A—C23—H23B	109.5
O2—C21—C11	113.94 (14)	H23A—C23—H23C	109.5
O5—C22—O6	124.31 (15)	H23B—C23—H23C	109.5
O5—C22—C7	122.51 (16)	C24—C23—H23A	109.5
O6—C22—C7	113.17 (14)	C24—C23—H23B	109.5
C18—O10—H10A	109.5	C24—C23—H23C	109.5
C2—C1—H1	119.8	N3—C24—C23	126.04 (17)
C6—C1—H1	119.8	N4—C24—N3	108.31 (16)
C6—C1—C2	120.38 (16)	N4—C24—C23	125.65 (17)
C1—C2—C19	120.38 (15)	N3—C25—H25	126.8
C3—C2—C1	119.79 (14)	C26—C25—N3	106.40 (17)
C3—C2—C19	119.76 (14)	C26—C25—H25	126.8
C2—C3—H3	120.1	N4—C26—H26	126.1
C2—C3—C4	119.79 (15)	C25—C26—N4	107.85 (16)
C4—C3—H3	120.1	C25—C26—H26	126.1
C3—C4—C5	120.21 (16)	C28—N5—H5A	125.5
C3—C4—C18	122.08 (15)	C30—N5—H5A	125.5
C5—C4—C18	117.69 (14)	C30—N5—C28	108.92 (16)
C4—C5—H5	119.8	C29—N6—H6A	125.6
C6—C5—C4	120.39 (15)	C30—N6—H6A	125.6
C6—C5—H5	119.8	C30—N6—C29	108.78 (16)
C1—C6—C17	120.59 (15)	H27A—C27—H27B	109.5
C5—C6—C1	119.38 (15)	H27A—C27—H27C	109.5
C5—C6—C17	119.97 (14)	H27B—C27—H27C	109.5
O7—C17—O8	125.41 (15)	C30—C27—H27A	109.5
O7—C17—C6	116.86 (14)	C30—C27—H27B	109.5
O8—C17—C6	117.73 (15)	C30—C27—H27C	109.5
O9—C18—O10	123.24 (16)	N5—C28—H28	126.4
O9—C18—C4	122.42 (15)	C29—C28—N5	107.12 (17)
O10—C18—C4	114.33 (14)	C29—C28—H28	126.4
O11—C19—O12	124.16 (14)	N6—C29—H29	126.5
O11—C19—C2	118.50 (15)	C28—C29—N6	107.06 (17)
O12—C19—C2	117.33 (14)	C28—C29—H29	126.5
C13—N1—H1A	125.1	N5—C30—N6	108.11 (16)
C13—N1—C14	109.87 (15)	N5—C30—C27	125.55 (18)
C14—N1—H1A	125.1	N6—C30—C27	126.33 (17)
			
C7—C8—C9—C10	1.5 (2)	C3—C4—C5—C6	−1.5 (2)
C7—C8—C9—C20	−176.91 (14)	C3—C4—C18—O9	−163.64 (16)
C8—C7—C12—C11	−1.9 (2)	C3—C4—C18—O10	17.1 (2)
C8—C7—C22—O5	1.9 (2)	C4—C5—C6—C1	1.6 (2)
C8—C7—C22—O6	−179.37 (14)	C4—C5—C6—C17	178.78 (14)
C8—C9—C10—C11	−1.2 (2)	C5—C4—C18—O9	14.6 (2)
C8—C9—C20—O3	−175.60 (15)	C5—C4—C18—O10	−164.63 (14)
C8—C9—C20—O4	5.4 (2)	C5—C6—C17—O7	167.31 (14)
C9—C10—C11—C12	−0.7 (2)	C5—C6—C17—O8	−13.4 (2)
C9—C10—C11—C21	177.89 (14)	C6—C1—C2—C3	−2.4 (2)
C10—C9—C20—O3	6.0 (2)	C6—C1—C2—C19	174.27 (14)
C10—C9—C20—O4	−173.05 (15)	C18—C4—C5—C6	−179.87 (14)
C10—C11—C12—C7	2.3 (2)	C19—C2—C3—C4	−174.28 (14)
C10—C11—C21—O1	−4.4 (2)	N1—C14—C15—N2	0.1 (2)
C10—C11—C21—O2	176.85 (15)	C13—N1—C14—C15	0.4 (2)
C12—C7—C8—C9	0.0 (2)	C13—N2—C15—C14	−0.5 (2)
C12—C7—C22—O5	−177.56 (16)	C14—N1—C13—N2	−0.8 (2)
C12—C7—C22—O6	1.2 (2)	C14—N1—C13—C16	177.78 (19)
C12—C11—C21—O1	174.23 (16)	C15—N2—C13—N1	0.8 (2)
C12—C11—C21—O2	−4.5 (2)	C15—N2—C13—C16	−177.7 (2)
C20—C9—C10—C11	177.28 (14)	N3—C25—C26—N4	0.0 (2)
C21—C11—C12—C7	−176.33 (14)	C24—N3—C25—C26	−0.4 (2)
C22—C7—C8—C9	−179.44 (14)	C24—N4—C26—C25	0.5 (2)
C22—C7—C12—C11	177.51 (14)	C25—N3—C24—N4	0.7 (2)
C1—C2—C3—C4	2.5 (2)	C25—N3—C24—C23	−179.16 (18)
C1—C2—C19—O11	−163.18 (15)	C26—N4—C24—N3	−0.7 (2)
C1—C2—C19—O12	15.8 (2)	C26—N4—C24—C23	179.13 (18)
C1—C6—C17—O7	−15.5 (2)	N5—C28—C29—N6	0.4 (2)
C1—C6—C17—O8	163.84 (14)	C28—N5—C30—N6	0.3 (2)
C2—C1—C6—C5	0.4 (2)	C28—N5—C30—C27	179.94 (17)
C2—C1—C6—C17	−176.77 (14)	C29—N6—C30—N5	0.0 (2)
C2—C3—C4—C5	−0.5 (2)	C29—N6—C30—C27	−179.67 (18)
C2—C3—C4—C18	177.76 (14)	C30—N5—C28—C29	−0.5 (2)
C3—C2—C19—O11	13.5 (2)	C30—N6—C29—C28	−0.2 (2)
C3—C2—C19—O12	−167.44 (15)		
